# Implications of Harvest on the Boundaries of Protected Areas for Large Carnivore Viewing Opportunities

**DOI:** 10.1371/journal.pone.0153808

**Published:** 2016-04-28

**Authors:** Bridget L. Borg, Stephen M. Arthur, Nicholas A. Bromen, Kira A. Cassidy, Rick McIntyre, Douglas W. Smith, Laura R. Prugh

**Affiliations:** 1 University of Alaska Fairbanks, Institute of Arctic Biology, 323 Murie Building, Fairbanks, Alaska 99775, United States of America; 2 National Park Service, Denali National Park and Preserve, P.O. Box 9, Denali Park, Alaska 99755, United States of America; 3 National Park Service, Yellowstone Center for Resources, Wolf Project, P.O. Box 168, Yellowstone National Park, Wyoming 82190, United States of America; 4 University of Washington, School of Environmental and Forest Sciences, Box 352100, Seattle Washington 98195, United States of America; Università degli Studi di Napoli Federico II, ITALY

## Abstract

The desire to see free ranging large carnivores in their natural habitat is a driver of tourism in protected areas around the globe. However, large carnivores are wide-ranging and subject to human-caused mortality outside protected area boundaries. The impact of harvest (trapping or hunting) on wildlife viewing opportunities has been the subject of intense debate and speculation, but quantitative analyses have been lacking. We examined the effect of legal harvest of wolves (*Canis lupus*) along the boundaries of two North American National Parks, Denali (DNPP) and Yellowstone (YNP), on wolf viewing opportunities within the parks during peak tourist season. We used data on wolf sightings, pack sizes, den site locations, and harvest adjacent to DNPP from 1997–2013 and YNP from 2008–2013 to evaluate the relationship between harvest and wolf viewing opportunities. Although sightings were largely driven by wolf population size and proximity of den sites to roads, sightings in both parks were significantly reduced by harvest. Sightings in YNP increased by 45% following years with no harvest of a wolf from a pack, and sightings in DNPP were more than twice as likely during a period with a harvest buffer zone than in years without the buffer. These findings show that harvest of wolves adjacent to protected areas can reduce sightings within those areas despite minimal impacts on the size of protected wolf populations. Consumptive use of carnivores adjacent to protected areas may therefore reduce their potential for non-consumptive use, and these tradeoffs should be considered when developing regional wildlife management policies.

## Introduction

Large carnivore conservation relies heavily on sustaining populations within protected areas [1], and protection within these regions provides the majority of viewing opportunities for these species [2]. The desire to see iconic, free ranging large carnivores is a driver for wildlife tourism around the globe and may improve acceptability of their presence by the general public and contribute to conservation goals ([3] but see [4]). However, large predators are wide-ranging and seldom confined within the boundaries of protected areas, creating difficult transboundary management issues. Outside and even inside of protected areas, conflict with humans is the single most important cause of mortality for large carnivores [5–7]. Yet the link between human-caused mortality of carnivores adjacent to protected areas and viewing opportunities within a protected region has not been evaluated quantitatively.

In North America, gray wolves (*Canis lupus*) are emblematic of management issues occurring at the borders of protected areas. Protection of wolves in National Parks, such as Yellowstone National Park (YNP) and Denali National Park and Preserve (DNPP), provides the opportunity for thousands of visitors to see wolves each year, but these wide-ranging carnivores often travel across park boundaries onto other public or private lands. Mortality of individual wolves from frequently viewed packs due to hunting or trapping outside these parks has sparked widespread controversy and prompted concern regarding the impact of these losses on population and pack dynamics. Although harvest (hunting or trapping) occurring outside park boundaries may not have population-level effects, harvest of particular individuals can lead to the decline or dissolution of entire packs [8,9]. If the packs or individuals most susceptible to harvest are those that provide the majority of viewing opportunities to visitors of protected areas, then harvest may influence wolf sightings even if harvest levels are too low to reduce population size. Similar impacts of harvest may affect carnivore sightings in other regions as well. In Africa, for example, the desire to see lions (*Panthera leo)* and cheetahs (*Acinonyx jubatus*) in their natural habitat is the main reason tourists visit the continent’s reserves, but these species are also the most vulnerable to threats such as human hunting adjacent to reserves [10].

The main objective of this study was to assess effects of harvest adjacent to protected areas on wildlife sightings, using wolves in Yellowstone National Park (YNP) and Denali National Park and Preserve (DNPP) as a case study. Agencies responsible for managing protected areas often have mandates to provide opportunities for visitor enjoyment. In the United States, the National Park Service is mandated to provide opportunities for visitor enjoyment of which wildlife viewing is an important component. Viewing large carnivores, particularly wolves and grizzly bears (*Ursus arctos*), is cited by visitors as one of the main reasons they come to YNP [11] and is a main indicator of a satisfying visitor experience in DNPP [12]. Additionally, in Alaska where wolves are among the most desired species for viewing [13], state wildlife management includes mandates to provide for multiple uses, including non-consumptive uses such as wildlife viewing [14]. In Montana, wildlife watching is listed by visitors and state residents as one of the primary activities in the state [15]. Wildlife viewing also brings an important socio-economic benefit to the states. Wolf watching activities in YNP following the reintroduction of wolves in 1995 brings an estimated $35 million annually to the states of Idaho, Montana and Wyoming [11]. Wildlife viewing is a driver of tourism for DNPP [16] and the state of Alaska [15,17] and wildlife viewing activities in Alaska supported over $2.7 billion dollars in economic activity in 2011 [17].At the same time, states are also mandated to provide for consumptive uses of wildlife, and harvest of wolves can provide significant economic benefits as well [18]. In 2011, statewide revenue in Montana from the purchase of wolf tags alone was over $400,000 [19] while hunting in Alaska supported over $1.3 billion dollars in economic activity [17].

**Fig 1:**
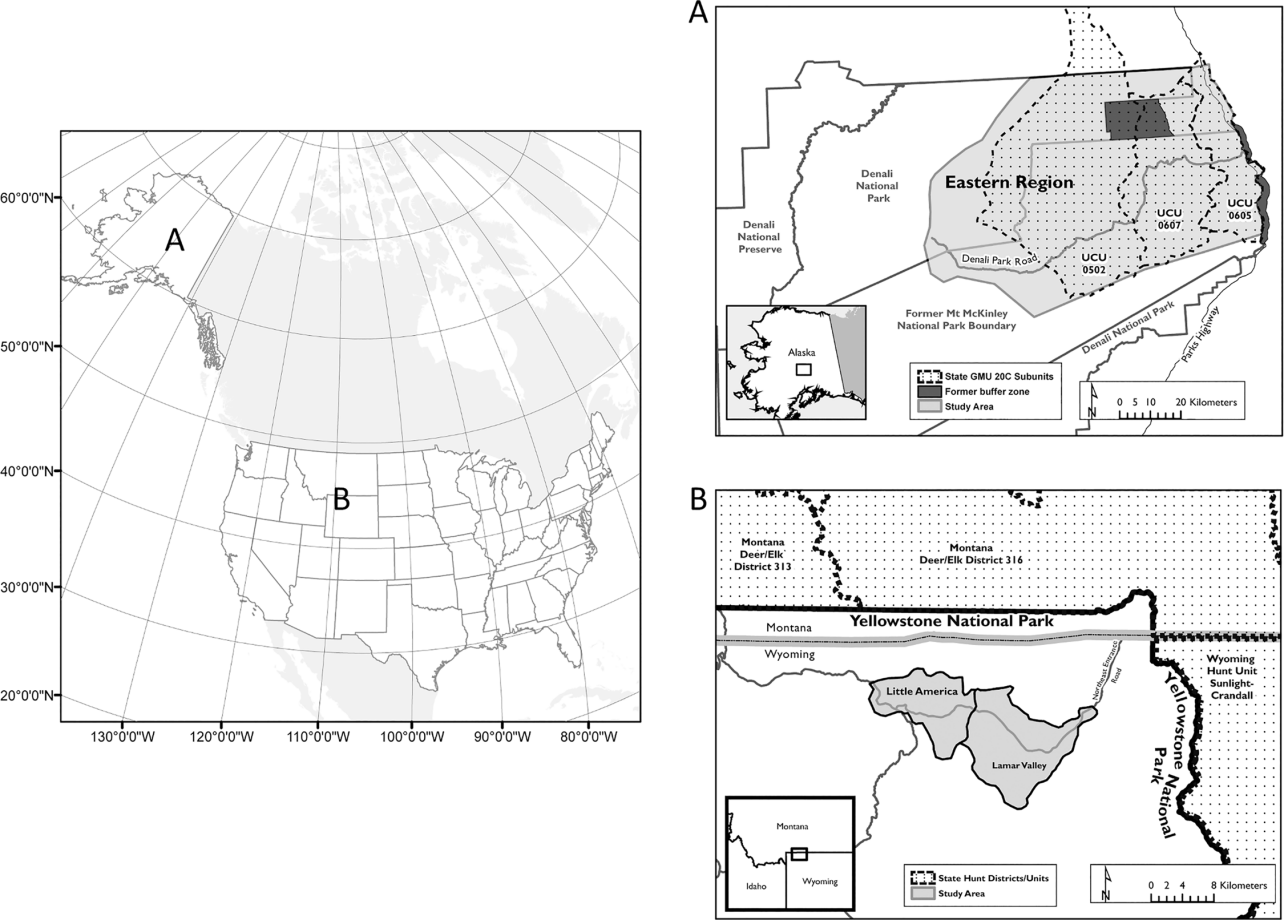
**Map of study areas for monitoring wolf sightings in the United States**: A) Denali National Park and Preserve study area with Uniform Coding Units (UCUs) within Game Management Unit 20C and former buffer zone where wolf hunting and trapping was prohibited from 2000 to 2010 shown and, B) Yellowstone National Park study area within the Northern Range with adjacent state hunt districts/units shown.

As part of the delisting process for gray wolves in Montana, Wyoming and Idaho, each state has developed wolf management plans that include wolf hunting seasons (for details on state management: www.westerngraywolf.fws.gov), prompting concern that hunting may impact wolf viewing opportunities in YNP [20]. In DNPP, a buffer zone prohibiting the trapping and hunting of wolves was established in key regions bordering DNPP from 2000 to 2010 (Fig 1). The buffer was abolished in March 2010 and viewing rates in DNPP subsequently declined [21], raising concerns that harvest of wolves near park boundaries might have been responsible.

To examine the effect of harvest on wolf sightings, we first examined levels of wolf harvest adjacent to each park and the composition of harvested wolves to determine whether breeding and collared wolves were more or less susceptible to harvest. Concurrent analyses showed that breeding wolves were more likely to be near the Denali Park Road than non-breeding wolves [22], indicating that breeding wolves may contribute disproportionately to sightings. However, we anticipated that less experienced (younger, non-breeding) wolves would be more likely to be harvested than the generally more experienced breeding wolves ([22], but see [23–25]). If this was the case, we expected that harvested wolves may be relatively unimportant to sightings, thereby reducing the potential effect of harvest on viewing opportunities. However, in YNP, the presence of radio-collars on wolves, regardless of breeding status, may increase sighting opportunities for visitors because NPS staff routinely scans for signals from collared animals to assist in locating and viewing wolves. Therefore, if there was disproportional harvest of collared wolves (regardless of breeding status), harvest could decrease viewing opportunities, especially in YNP.

We analyzed data on wolf sightings, pack sizes, den site locations, and harvest adjacent to DNPP from 1997–2013 and YNP from 2008–2013 to evaluate the relationship between harvest of wolves and wolf viewing opportunities. We hypothesized that changes in wolf population size and den site proximity to park roads are the main drivers of wolf sightings and that additionally, the presence of harvest (or absence of the harvest buffer) would reduce wolf sightings. Alternatively, changes in wolf population size and den site proximity to park roads could be the main drivers of wolf sightings, and harvest could have comparably negligible effects.

## Methods

### Study areas

Our study area encompassed two national parks in North America (Fig 1, Table 1). The DNPP study area encompassed 6,350 km^2^ of the eastern region of the park and adjacent areas north of the Alaska Range (Fig 1). Elevation ranges from 150–3,000 m and contains habitat patches of boreal forest, high alpine, braided rivers, and willow-lined creeks. The diversity of habitat types supports populations of caribou (*Rangifer tarandus*), Dall’s sheep (*Ovis dalli*), and moose (*Alces alces*) which constitute the main prey base for wolves in the region. The YNP study area encompassed approximately 1,000 km^2^ of the Northern Range within and adjacent to the park (Fig 1). Elevation ranges from 1,500–2,400 m, with lower elevations characterized by large open meadows and shrub steppe vegetation and higher elevations characterized by coniferous forests [26]. Elk (*Cervus elaphus*) are the main prey for wolves in this region, but wolves also prey secondarily on mule deer (*Odocoileus hemionus*), white-tailed deer (*O*. *virginianus*), and bison (*Bison bison*).

**Table 1:** Metrics summarizing wolf sighting datasets in Denali (Denali National Park and Preserve, Alaska, USA) and Yellowstone (Yellowstone National Park, Wyoming, USA). Table entries for wolf population size, road pack population size, number of road packs (packs whose home range overlapped park roads), and the annual probability of sighting are mean values, with the range among years in parentheses.

Metric	Denali	Yellowstone
Study Period	1997–2013	2008–2013
Length of road	88.5 km	42.3 km
Relevant Harvest Periods	Area closed to harvest adjacent to park: 2000–2010	Harvest Open: Idaho and Montana: 2009, 2011, 2012 Wyoming: 2012
Hunting Season	Mid-August to end of April or May	Varied by state
Hunting Limits	Bag limit range: 5 to 10 wolves	Varied by state
Trapping Season	November 1 to April 30	Varied by state
Trapping Limits	No bag limits	Varied by state
Wolf Population Size	40.8 (23–74)	45.7 (33–84)
Road Pack Population Size	32.8 (12–47)	27.4 (12–43)
Number of Road Packs	5.4 (3–9)	3.1 (2–5)
Annual Probability of Sighting	00.21 (0.04–0.45)	0.70 (0.45–0.85)

### Data collection

#### Population and pack counts

Biologists have radio-collared wolves in the DNPP study region since 1986 [27] and within YNP since the reintroduction of wolves in 1995 [28]. Each year, 6–22 wolves from 10–20 packs were fitted with radio collars in DNPP [29] and 10–20 wolves from 5–12 packs were collared in YNP ([28], see [29] for handling protocols). Wolf project staff in both YNP and DNPP used a combination of aerial and ground monitoring techniques to collect data on wolf locations, numbers of pack members, pack composition, active den site locations and use, breeding status of individual wolves and timing and suspected causes of mortality [27,30]. Capture and handling protocols were approved by the National Park Service Institutional Animal Care and Use Committee and were in accordance with recommendations from the American Society of Mammalogists [31]. Work was conducted under annual National Park Service permits, annual State of Alaska Department of Fish and Game scientific permits, and the University of Alaska permit (253217–3).

#### Harvest

All areas outside the DNPP boundary were open to hunting and trapping under state regulation, with the exception of a closed area established by the Alaska Board of Game in 2000, expanded in 2001 and 2002 (Fig 1), and abolished in 2010. Although the closed area was relatively small (75 km^2^ in 2000, 233 km^2^ from 2002–2010), it included areas that supported high seasonal densities of caribou and associated wolf activity [27]. In Game Management Units (GMU) 20A and 20C adjacent to the park’s boundaries, the hunting season ranged from mid-August to the end of April or May with a bag limit ranging from 5–10 wolves, and the trapping season spanned November 1– April 30 with no bag limits for either unit. Subsistence and sport hunting and trapping were permitted in the Preserve and new park additions of DNPP, but all harvest was prohibited in the area of the original Mt. McKinley National Park (Fig 1). Outside YNP, wolves were hunted in 2009, 2011 and 2012 in Idaho and Montana, and in 2012 in Wyoming, with open seasons and limits that varied among hunting units within states. Wolves were not harvested in 2010 due to relisting under the Endangered Species Act. The numbers of wolves harvested from regions adjacent to park boundaries were obtained from state harvest records and mortality of collared wolves.

#### Harvest of collared and breeding wolves

To examine whether collared and breeding wolves were harvested disproportionately, we used chi-squared and Fisher exact tests to compare the proportion of collared and breeding wolves harvested in areas surrounding each park with their proportions in each park population. In DNPP, we used mortality records to determine the number of collared wolves that were shot or trapped in Uniform Coding Units (UCU) adjacent to DNPP (UCUs 605, 607, and 502) from 1996 to 2012 (Fig 1). We included all recorded wolf harvest within UCUs 605 and 607 in analyses because these UCUs were within the buffer zone or immediately adjacent to DNPP (Fig 1). UCU 502 extended north beyond DNPP and we therefore attempted to include only instances of wolves harvested in UCU 502 that occurred within the former buffer zone using information on the location of harvest. Instances of harvest with unknown locations within UCU 502 were included in the count of harvested wolves in the region. In YNP, we consulted with state agencies to estimate the number of collared and/or breeding wolves and the total number of wolves harvested outside of YNP that were from packs that lived predominantly in YNP. Harvested wolves that were uncollared were judged to have originated from YNP packs if the ages, colors, and sexes matched wolves recently missing from YNP.

We pooled data across years with wolf harvest (1996–2012 for DNPP and 2009, 2011, and 2012 for YNP). We calculated the proportion of collared wolves in the population as the number of individuals collared in or before year *t* that were still alive by August of year *t* divided by the fall population estimate. Similarly, we determined the proportion of breeders in the population as the number of collared individuals identified as breeders divided by the fall population estimate. We restricted our analysis to collared breeders because identification of uncollared breeders in the harvest was not always possible. We determined the proportion of collared or breeding wolves in the harvest as the number of collared/breeding wolves harvested divided by the number of wolves harvested in surrounding UCUs (DNPP) or from YNP packs.

#### Sighting data

Each study area is bisected by a road (Denali Park Road in DNPP and Northeast Entrance Road in YNP, Fig 1) providing visitor access to the region and wolf viewing opportunities. Traffic along the portion of the road where wolf observations were collected in DNPP was limited to 10,512 vehicle trips per summer season as per DNPP management plans [32]. Although there were slight variations, the traffic was essentially kept at a consistent level for the duration of the study period. According to traffic counts from the north and northeast entrance stations at YNP, traffic into the park gradually increased during the study period [33].

#### DNPP

We used data on wildlife sightings along the Denali Park Road collected during bus trips into the park from the Savage River entrance station at mile 15 (24.1 km) to Eielson Visitor Center at mile 66 (106.2 km) from 1997–2013. Data were collected by bus drivers as written observations or on panels installed on buses and by park staff as written observations or on handheld devices. Observers recorded all sightings of wolves during all westbound trips (see S1 Appendix for more details).

#### YNP

From 2008 to 2013, YNP staff (R. McIntyre) traveled through the Lamar Canyon and Little America region (Fig 1) every morning (from approximately 0430 or 0500 to 1100 or 1200 hours) and consistently recorded all direct sightings of wolves. These 6 years represent a sample of years with and without harvest, consistent monitoring of sightings, and a relatively stable wolf population. We reviewed the daily field notes and recorded the start and end time of each daily observation period and attributes of every wolf sighting (location and duration of sighting, number of wolves seen, pack affiliations) in June, July and August.

#### Annual probability of sightings metric

We calculated the annual probability of sighting metric in DNPP as the proportion of bus trips where at least one wolf was seen (S1 Table). In YNP, we calculated this metric as the number of days with direct sightings of wolves in Lamar Valley or Little America (Fig 1) divided by the number of days in the observation period (i.e. number of days in June, July and August), corrected for effort:

$$YNP\;P_{sighting}=\frac{S_{t}}{O_{t}}\times \frac{E_{t}}{E_{max}}$$

where S_t_ is the number of days with sightings in year *t*, O_t_ is the number of days in the observation period, E_t_ is the hours of effort in year *t*, and E_max_ is the maximum number of hours in the field from sampled years (S2 Table).

We predicted that the annual probability of sighting for a wolf was positively related to wolf population size and den site proximity to the roads and negatively related to the number of wolves or breeders harvested. We examined 2 metrics of population size: spring estimates of total wolf population size in each study area (TotalPop), and a metric that combined the estimated size of packs whose home range overlapped park roads (road packs) with distances from den sites to the nearest road (the Pack Near Road Index, or PNRI, Table 2). TotalPop represented a simple and potentially useful metric that could be calculated in spring prior to denning while PNRI was a metric that combined a spatially-explicit measure (den site distance from the road) with a population measure (road pack size). We initially investigated a separate covariate for road pack size alone (S1 and S2 Figs, S6 Table) and found that the metric that combined road pack size and den distance (PNRI) explained more variance in sightings. We therefore used PNRI in our final model set.

**Table 2:** Explanatory variables used to model annual probability of sighting rates in Denali National Park, Alaska, USA. Prediction column describes the predicted change in the response variable (annual probability of sighting) to an increase in the explanatory variable.

Variables	Description	Prediction
***Wolf Population***		
TotalPop	Spring estimates of total wolf population in each study area	Increase
PNRI	Pack Near Road Index. Metric combining the estimated size of road packs with distances of pack den sites to road	Increase
***Wolf Harvest***		
WolfHarv	Number of wolves harvested adjacent to park boundaries prior to sighting year	Decrease
BreedHarv	Binary, if a breeding wolf from a road pack was harvested in year prior to sighting year	Loss of breeding wolf: decrease
Buffer	Binary, presence or absence of hunting and trapping buffer zone	Presence of buffer zone: increase

TotalPop was obtained by compiling spring wolf pack counts for packs in each study area. We used ArcGIS 10.0 (Environmental Systems Research Institute, Redlands, CA) to assess home range overlap with park roads. PNRI was calculated using pack size and den site distance for road packs. Wolf management plan objectives require closing areas around known den sites to hikers [34]. Thus, den site locations and use were closely monitored for wolf packs in areas along the road corridors. We determined the distance of den sites to the nearest location on the road using the “near” tool in ArcGIS version 10.2 (ESRI 2011, ArcGIS Desktop: Release 10. Redlands, CA: Environmental Systems Research Institute). For all road packs in the sighting year, we divided the pack size by the distance from the pack’s den or rendezvous site to the nearest road and defined the PNRI as the sum of these measures for all packs in the sighting year. In cases where there was more than one den or rendezvous site used by a single pack, we used the mean of the distances of multiple den or rendezvous sites as the value for that pack. Thus, an increase in pack sizes or numbers of packs, or a decrease in distances of pack activity centers from the road, would cause PNRI to increase.

For DNPP, we evaluated three metrics describing wolf harvest: number of wolves harvested in the region (WolfHarv), harvest of breeding wolves (BreedHarv) and the presence/absence of a wolf trapping buffer (Buffer) located outside of DNPP (Fig 1).WolfHarv was the number of wolves harvested in Uniform Coding Units (UCUs) 605 and 607 (Fig 1) in the regulatory year prior to the sighting year (July 1 of year t-1 to June 30 of year t). BreedHarv was a binary factor describing if a breeding wolf from a road pack was harvested prior to the sighting year. The trapping buffer was present from 2000–2010 and absent 1997–1999 and 2011–2013 (Table 1). In YNP, we obtained information on the number of wolves harvested outside of YNP from Yellowstone Wolf Project staff in collaboration with state wildlife agency professionals in Montana, Wyoming, and Idaho.

### Effect of harvest on sightings

We evaluated factors that influenced annual wolf sightings in DNPP using a suite of generalized linear models and Akaike information criterion corrected for sample sizes and an estimate of overdispersion (QAICc) to rank models [35]. We used the glm function in Program R (R Core Team 2013) to model wolf sightings using a binomial distribution with the response variable as the annual probability of wolf sightings, weighted by the number of trips per year to account for sample size. Predictor variables consisted of the 2 population and 3 harvest metrics described above (Table 2), and our model set consisted of 14 models selected a-priori that included 1–3 predictors per model (Table 3). We used the MuMIn package in R [36] for model selection and derived untransformed parameter estimates and associated standard errors from the top ranked model.

**Table 3:** Candidate model set and model selection criteria evaluating factors potentially affecting probability of wolf sightings along Denali Park Road in Denali National Park and Preserve, Alaska, USA.

Model	K^a^	QAICc	ΔQAICc	Model Likelihood	QAICc Weight
PackNearRoad^b^+Buffer^c^+WolfHarv^d^	4	41.70	0.00	1.00	0.33
PackNearRoad+Buffer	3	42.15	0.44	0.80	0.27
PackNearRoad	2	43.42	1.71	0.43	0.14
PackNearRoad+WolfHarv	3	44.68	2.98	0.23	0.07
Buffer	2	45.92	4.22	0.12	0.04
TotalPop^e^+Buffer	3	45.95	4.25	0.12	0.04
PackNearRoad+Buffer+BreedHarv^f^	4	46.13	4.43	0.11	0.04
PackNearRoad+BreedHarv	3	46.55	4.85	0.09	0.03
TotalPop+Buffer+WolfHarv	4	47.84	6.14	0.05	0.02
TotalPop+Buffer+BreedHarv	4	47.92	6.21	0.04	0.01
TotalPop+BreedHarv	3	49.17	7.47	0.02	0.01
TotalPop	2	50.77	9.07	0.01	0.00
TotalPop+WolfHarv	3	54.10	12.40	0.00	0.00
WolfHarv	2	59.19	17.49	0.00	0.00

^a^ Number of parameters in the model

^b^ Pack Near Road Index

^c^ Buffer is a factor indicating the presence/absence of a wolf hunting and trapping buffer

^d^ WolfHarv is the number of wolves harvested in the prior year

^e^ TotalPop is the population size

^f^ BreedHarv is a factor indicating if breeders were or were not harvested from road packs in the prior year.

We used a variance partitioning procedure to quantify how much of the variation of the top-ranked model was explained by the pure effect of each explanatory variable and the interaction of the variables [37–39]**.** We compared estimates of population size between years with and without the buffer zone using a one-tailed t-test. We used nonparametric Mann-Whitney-Wilcoxon tests to compare PNRI and annual probability of sightings between these periods because these variables did not meet the assumptions of t-tests.

We lacked sufficient years of data in YNP to construct quantitative models of sightings including all covariates. Therefore, we visually examined patterns in the annual sighting metric in relation to TotalPop and PNRI. We compared annual probability of sightings in years with and without harvest of wolves from packs in the prior regulatory year using a one-tailed t-test.

## Results

### Harvest of collared and breeding wolves

#### DNPP

Wolves were harvested on state land adjacent to DNPP in 16 of the 17 years in our dataset (1996–2012). Across all 17 years, on average 5 (SD 3.5) wolves were harvested each year (S3 Table). Pooled across all years with harvest, neither the proportion of collared wolves in the harvest (0.25) nor the proportion of known (collared) breeding wolves in the harvest (0.16) were significantly different than expected given their frequency in the population (collared wolves in population: 0.29, χ2 = 0.610, df = 1, *P* = 0.44, collared breeders in population: 0.17, χ2 = 0.072 df = 1, *P* = 0.79).

#### YNP

In 2009, 4 park wolves were harvested from the study area. In 2011, 2 wolves ranging primarily within YNP but not considered members of a road pack were shot close to the park boundary. In 2012, 9 wolves that primarily lived within the Northern Range study area were harvested and a total 12 wolves that lived in the entire YNP were harvested. The proportion of collared wolves in the harvest (0.53) was greater than expected given the proportion of collared wolves in the Northern Range population (0.24, Fisher’s exact test: *P* = 0.03). Similarly, in the entire YNP region, the proportion of collared wolves in the harvest (0.56) was greater than expected given the proportion of collared wolves in the YNP population (0.26, Fisher’s exact test: *P* = 0.01, S4 and S5 Tables). The proportion of collared breeding wolves in the harvest (0.21) was not significantly different than the proportion of collared breeders in the Northern Range (0.17, 2-sided fisher’s exact test, *P* = 0.37).

### Annual Probability of Sighting

#### DNPP

We used sighting data from 2062 trips along the Denali Park Road from 1997–2013. One or more wolves were observed on 307 of the 2062 trips (S1 Table). Both the number of wolves denning near the road and wolf harvest influenced the mean probability of viewing wolves in DNPP. The top ranked model included the Pack Near Road Index (PNRI), the presence of the wolf harvest buffer, and the number of wolves harvested (Table 3). The number of wolves denning near the road was positively associated with the probability of viewing wolves (Table 4). The presence of the buffer was also positively associated with the probability of viewing wolves. The number of wolves harvested in the prior year was negatively associated with the probability of viewing a wolf, although the effect was not significant as the confidence intervals overlapped zero (Table 4).

**Table 4:** Model-averaged parameter estimates for annual probability of sighting model evaluating factors potentially affecting probability of wolf sightings along Denali Park Road in Denali National Park and Preserve, Alaska.

	β	SE	95% CL	
			Lower	Upper
(Intercept)	-2.70	0.488	-3.660	-1.748
PNRI^a^	22.84	8.455	6.264	39.408
Buffer (Presence)^b^	0.96	0.448	0.082	1.838
WolfHarv^c^	-0.10	0.057	-0.211	0.013

^a^ PNRI is the Pack Near Road Index

^b^ Buffer is the presence of a wolf hunting and trapping buffer

^c^ WolfHarv is the number of wolves harvested in surrounding regions.

The pure effects of PNRI, the presence of the buffer, and the number of wolves harvested in the prior year explained 53%, 42.3%, and 15.1%, respectively, of the variation in the top-ranked model. The combined effect of the variables PNRI, buffer presence, and the number of wolves harvested in the prior year explained the largest proportion of variation in the top-ranked model (61.7%).

**Fig 2:**
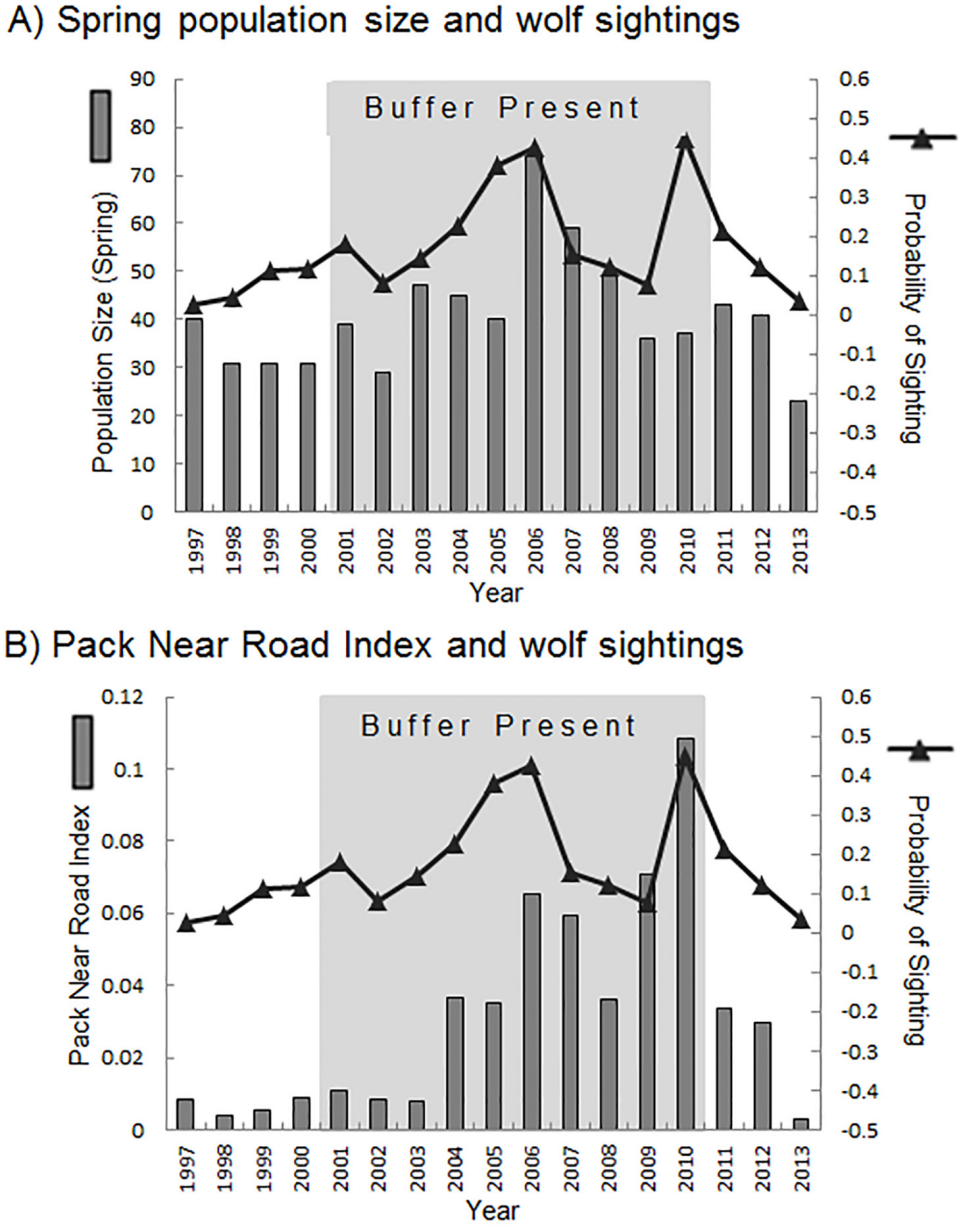
**Probability of wolf sighting along the Denali Park Road from 1997 to 2012 (black triangles)** in relation to A) spring population size (gray bars) and B) the Pack Near Road Index (number of wolves in road packs divided by den distances from the road, gray bars) in Denali National Park and Preserve, Alaska, USA. Shaded areas indicate the time period (2000–2010) when a harvest buffer zone adjacent to the park was in effect.

**Fig 3:**
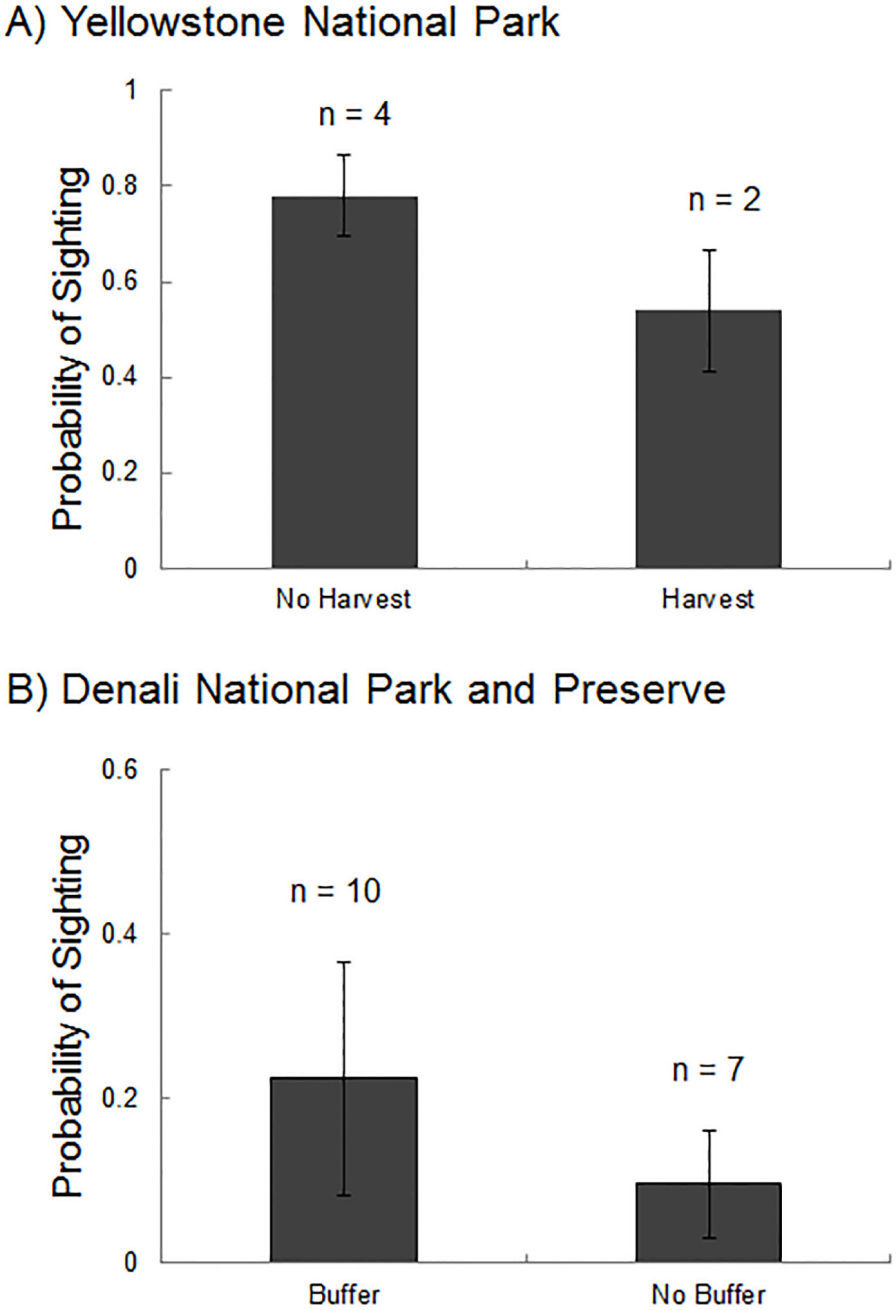
**Mean probability of sighting for wolves** A) in Lamar Valley and Little America following years with and without harvest of pack wolves, Yellowstone National Park, Wyoming, USA and B) along the Denali Park Road following years with and without the presence of a buffer zone prohibiting the trapping and hunting of wolves outside of Denali National Park and Preserve, Alaska, USA. Standard error bars and sample sizes (number of years) are shown.

The annual probability of sighting appeared to roughly follow the trend of the annual PNRI and spring population size, with peaks in sightings coinciding with peaks in either PNRI or total population size (Fig 2, see S1 Fig for figure with road pack size). Population size, PNRI and the probability of sighting were significantly higher in years when the buffer zone was in place (Table 5, Fig 3).

**Table 5:** Comparisons of the annual probability of wolf sighting, wolf population, and Pack Near Road Index (PNRI) for years following the presence (2001–2010) and absence (1997–2000, 2011–2013) of a hunting and trapping buffer adjacent to Denali National Park and Preserve, AK, USA. Table entries are the mean values (SE), test statistics (*t* for t-test and *W* for Mann-Whitney-Wilcoxon test), and associated probability for each metric.

	Buffer	No Buffer	Test Stat	*P*-value
Population	45.5 (4.11)	34.3 (2.73)	t_15_ = -2.27	0.039
Sightings	0.22 (0.045)	0.10 (0.025)	*W =* 57	0.033
PNRI	0.04 (0.010)	0.01 (0.005)	*W* = 60	0.014

#### YNP

**Fig 4:**
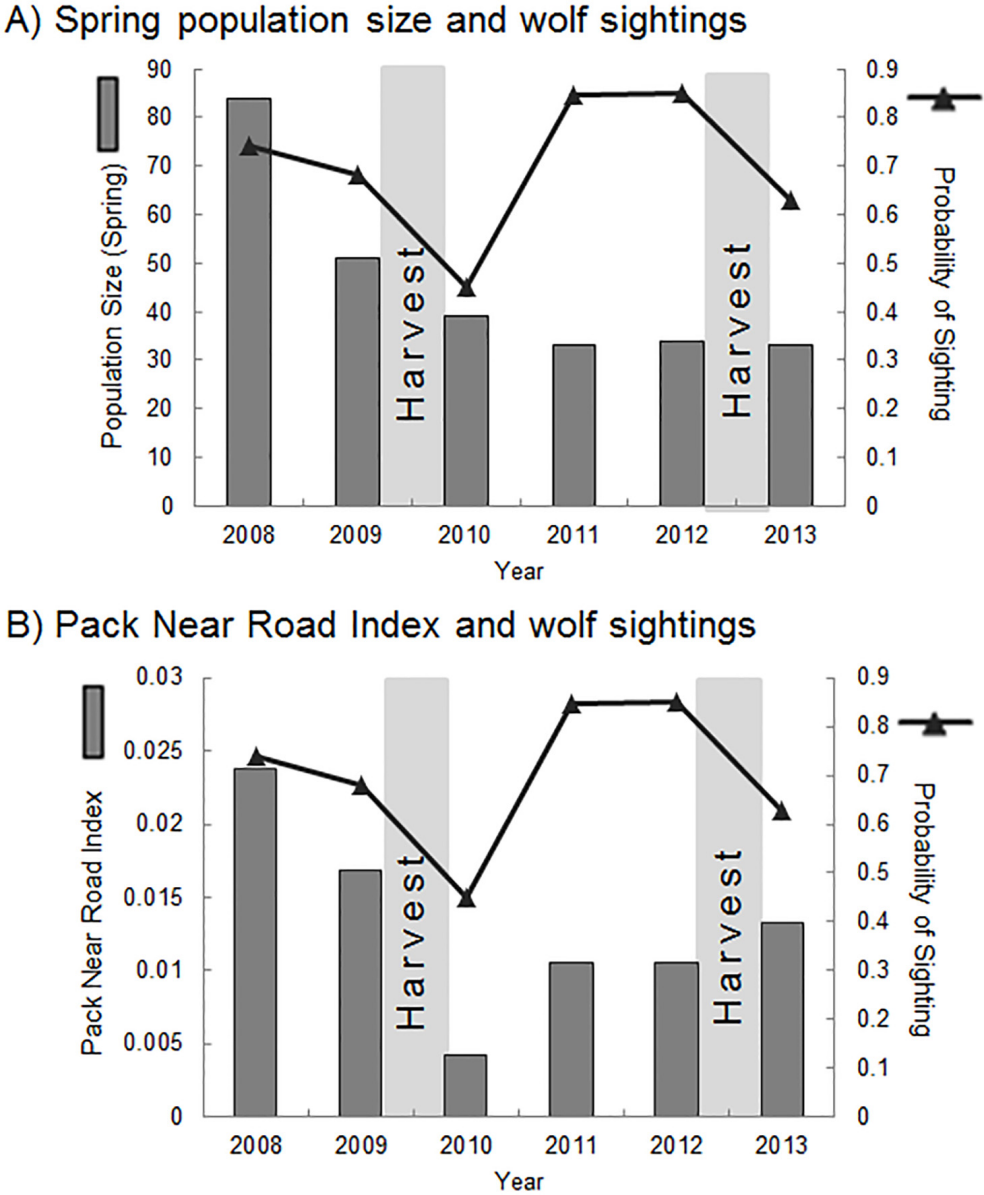
**Probability of wolf sighting in Little America and Lamar Valley from 2008–2012 (black triangles)** in relation to A) spring population size and B) Pack Near Road Index (number of wolves in road packs divided by den distances from the road) in Yellowstone National Park, Wyoming, USA. Shaded areas indicate years following harvest of wolves from packs. Two non-pack wolves were harvested prior to 2012.

We used sighting data from 552 days in YNP from 2008–2013. One or more wolves were observed during 436 of the 552 days (S2 Table). There were 2 years of sighting data following harvest from YNP road packs (2010 and 2013) and 4 years with no prior road pack harvest (2008, 2009, 2011 and 2012). The annual probability of sighting metric for YNP appeared to roughly mirror spring population size and PNRI, but sightings were lower in years following harvest of wolves from road packs than in years with similar population size (Fig 4, see S2 Fig for figure with road pack size). The mean probability of sighting was lower following years with harvest of road pack wolves (0.54 ± 0.127 SE) than in years without harvest of a road pack wolf (0.78 ± 0.084 SE, *t*_4_ = 2.88, *P* = 0.02, Fig 4). If we consider 2012 as a post-harvest year (based on the harvest of 2 non-road pack wolves in 2011), the mean probability of sighting was not significantly different between years following harvest (0.64 ± 0.040 SE) and years without harvest (0.76 ± 0.086 SE, *t*_4_ = 0.92, *P* = 0.21).

## Discussion

This study provides the first quantitative evidence that harvest of wildlife adjacent to protected areas can reduce wildlife sighting opportunities. Harvest of wolves was associated with reduced sightings in both Denali and Yellowstone National Parks. The probability of viewing a wolf was 45% greater in YNP following years with no harvest of a wolf from a road pack, and sightings in DNPP were more than twice as high in years with the presence of a wolf harvest buffer (Fig 4). There was a trend indicating that sightings decreased as the number of wolves harvested adjacent to DNPP increased, although the relationship was weak. These findings imply a trade-off between harvest (i.e., consumptive use) of large carnivores and the non-consumptive viewing opportunities and associated economic benefits. Additionally, we found that population size, pack size and den site location were strong drivers of sighting opportunities for wolves within these protected areas. These findings suggest that harvest is likely to have particularly strong effects on sightings when harvest reduces population size or affects breeding behavior within protected regions.

Human-caused mortality of large carnivores adjacent to protected areas can lead to population declines within the protected region [40–42] which our research indicates has the largest potential to decrease viewing opportunities. Although harvest of wolves in our study systems may not have occurred at rates generally considered sufficient to reduce population size (reviewed in [43]), harvest may influence sightings through other mechanisms. Behavioral avoidance of humans by wolves following exposure to hunting or trapping could reduce sightings. Although wolves show preference for linear travel corridors [44] and roads with low levels of traffic [8,45], wolves will avoid of high levels of human activity [46–48]. The presence of hunters is known to affect large carnivore behavior and movements [49]. However, the direct link between exposure to harvest and subsequent behavioral avoidance leading to reduction in sightings was not explicitly tested in our analysis and warrants further investigation. Monitoring behavior of large carnivores that survive negative encounters with humans is needed to determine the strength of these anti-predatory responses.

Selection for behavioral traits may be another method by which harvest of carnivores could decrease sightings. In our study systems, a small number of wolves may contribute to a large number of wolf sighting opportunities. Harvest can selectively target ‘bold’ individuals [50, 51], thereby removing bold individuals and over time, the trait, from populations. Indeed, phenotypic changes driven by human harvest can outpace selection of traits driven by other forces [52]. As large carnivores that are less wary may contribute disproportionately to viewing opportunities, sightings could decrease if harvest selects these individuals.

We hypothesized that harvest of breeding wolves would disproportionately influence sightings, because these individuals play an important role in pack continuity and reproduction [9, 53] and were more likely to be near the road than non-breeding wolves [22]. Although harvest reduced sightings, the breeding status of harvested wolves was not identified as an important factor in our analyses (Table 1). Instead, our results suggest that harvest of wolves from road packs may have a larger influence on sightings than harvest of other wolves. Sightings were not reduced in YNP following the harvest of 2 wolves that were not members of road packs. These wolves resided in the park but likely contributed little to sightings as they did not live along the road corridor. However, we caution that our results from YNP were based on a limited sample size. We recommend continued monitoring of carnivore sightings and increased emphasis on identifying age, reproductive status and social group affiliation for carnivores harvested adjacent to protected areas to increase our understanding of these influences on sightings.

Collared wolves made up over half of the harvest adjacent to YNP but were only approximately a quarter of wolves in the YNP population, whereas collared wolves were harvested in proportion to their occurrence in the DNPP population. A major difference between these parks is that harvest near YNP is through hunting whereas harvest near DNPP is primarily through trapping. Although both harvest methods have the potential to act as selective forces on behavioral traits (i.e. bold or unwary individuals), hunting involves more active selection by humans whereas trapping passively selects wolves. This distinction could explain why there was disproportional harvest of collared wolves adjacent to YNP and not adjacent to DNPP if hunters targeted collared wolves. It is important to note that results from YNP were based on three years of data, and longer term analysis could yield different results. Still, the disproportional harvest of collared individuals may be a mechanism by which sightings decrease following harvest, as the presence of collared individuals aids in locating individuals (R. McIntyre, pers. obs.) or understanding behavioral patterns [54] thereby creating viewing opportunities.

In both parks, the number of identified breeders that were harvested was not different than expected given their proportion in the population. We expected that breeders would be less likely to be harvested, particularly when trapping was the primary source of harvest, as in DNPP [23]. It is possible that the benefit of experience and age in avoiding trapping may be offset in protected regions by habituation to human activity and use of linear travel corridors during the summer months [8]. Given that the primary source of harvest was hunting, the result in YNP is consistent with previous findings [23–25].

The presence of the trapping and hunting buffer zone was associated with increased wolf sightings in DNPP. Both the wolf population size and PNRI, which were strongly associated with increased wolf sightings, were also greater during the period when the buffer zone was in place. Thus, the presence of the buffer may have influenced local population size and the likelihood that wolves would den near the park road. Alternatively, the increase in sightings may have been a result of coincidental peaks in population size or PNRI as a result of variables not measured or explicitly included in our models. Two variables generally considered to be strong drivers of wolf population dynamics are prey density and snow conditions, which influence prey vulnerability to wolf predation [27]. However, during the period of the study, prey densities were relatively consistent [55–57]. Similarly, although snow conditions varied among years, there has been no statistically significant trend in the annual snowfall data for park headquarters over the past 20 years [58]. Traffic levels, managed at a consistent level during the study period, likely did not influence annual trends in sightings. Similarly in YNP, there was a decrease in sightings during years with harvest that did not appear to be explained by a change in wolf population size or change in the size of packs near the road (Figs 3 and 4). Although our sample size was low, the decrease was statistically significant. Neither climatic conditions nor prey base were thought to significantly alter wolf population dynamics in YNP during the study period. The elk population was stable during the study time period, and although snow depth in winter 2010–2011 was above average, the other winters were within the average range for snowfall and temperature [59]. Although there was an increase in visitation in YNP during the study period, there was no indication that annual wolf sighting trends were influenced by this pattern in visitation [33].

The opportunity to view free ranging large carnivores is an important driver for wildlife tourism worldwide, and the National Park Service mission in particular emphasizes the preservation of wildlife resources in their natural condition for the non-consumptive benefit and enjoyment of the public. Thus, factors that influence sightings of iconic wildlife such as wolves are important to track and understand. Here, we have shown that consumptive use of a large carnivore reduces opportunities for non-consumptive use in protected areas. Limiting harvest of large carnivores along the boundaries of protected areas may provide a strategy to increase sighting opportunities for visitors to these areas and the associated economic benefits to adjacent communities. However, there are associated costs of limiting harvest, given the revenue generated from hunting [17, 19, 60] and the potential of harvest to reduce threats to livestock and increase land owner’s acceptance of large carnivores [61, 62]. Cross boundary movements will continue to make large carnivore management an on-going source of debate. Wolf viewing and harvest opportunities are 2 of the many issues surrounding cross boundary wolf management. There are many stakeholders, including state and federal management agencies, private land owners, trappers, hunters, non-profit agencies, environmental advocates, and the general public Effective management in areas where cross boundary movements are common requires knowledge of complex system dynamics, in addition to understanding and defining the objectives of stakeholders, and quantifying the associated costs and benefits of management actions.

## Supporting Information

S1 Appendix Recording Wildlife Sightings in Denali National Park and Preserve. (DOCX)

S1 Fig Wolves in road packs and the probability of wolf sightings along the Denali Park Road, Alaska, USA. Cumulative count of wolves in road packs in the eastern region of Denali National Park and Preserve (grey bars) and the probability of wolf sightings along the Denali Park Road (black triangles) from 1997 to 2012. Shading indicates years with a harvest buffer zone adjacent to the park in effect.
(TIF)

S2 Fig Wolves in road packs and the probability of wolf sightings in Yellowstone National Park, Wyoming, USA. Cumulative count of wolves in road packs in the Northern Range of Yellowstone National Park (grey bars) and probability of wolf sightings in Little America and Lamar Valley (black triangles) from 2008–2012. Hashed bars indicate years preceded by harvest of wolves from road packs. Light gray shading indicates years preceded by harvest of non- pack wolves.
(TIF)

S1 Table Annual probability of sighting index for Denali National Park and Preserve, Alaska, USA. Sample size (in number of trips), number of trips with wolf sightings, and annual probability of sighting index for wolves along the Denali Park Road from 1997 to 2013.
(DOCX)

S2 Table Annual probability of sighting index for Yellowstone National Park, Wyoming, USA. Sample size (in number of days within the observation period), number of days with wolf sightings, relative effort for each year (calculated as hours of effort in the given year divided by the maximum number of hours in the field from sampled years), and annual probability of sighting index for wolves in the Lamar Valley and Little America region of Yellowstone National Park from 2008 to 2013.
(DOCX)

S3 Table Summary of wolf harvest for the Eastern Region of Denali National Park and Preserve, Alaska, USA. Population size estimates, number of collared wolves, number of collared breeding wolves, and their proportions in the population and harvest included. Population size, number of collared wolves, and number of collared breeders were pre-hunt numbers.
(DOCX)

S4 Table Summary of wolf harvest for Northern Range packs (including Mollie’s pack) in Yellowstone National Park, Wyoming, USA. Population size estimates, number of collared wolves, number of collared breeding wolves, and their proportions in the population and harvest for Northern Range packs (including Mollie’s pack) included. Population size, number of collared wolves, and number of collared breeders were pre-hunt numbers.
(DOCX)

S5 Table Summary of wolf harvest for wolf packs in Yellowstone National Park, Wyoming, USA. Population size estimates, number of collared wolves, number of collared breeding wolves, and their proportions in the population and harvest included. Population size and number of collared wolves were pre-hunt numbers.
(DOCX)

S6 Table Model selection table evaluating factors potentially affecting probability of wolf sightings in Denali National Park and Preserve, Alaska, USA (including the factor RoadPop). Candidate model set includes the factor RoadPop. K is the number of parameters in the model, PNRI is the Pack Near Road Index, TotalPop is the wolf population size, RoadPop is the number of wolves in packs that overlap the Denali Park Road, Buffer is a factor indicating the presence/absence of a harvest buffer, WolfHarv is the number of wolves harvested in the prior year and BreedHarv is a binary factor describing if breeders were or were not harvested from road packs in the prior year.
(DOCX)
