# Supplementary material for: Implications of Harvest on the Boundaries of Protected Areas for Large Carnivore Viewing Opportunities
Source: PLoS One. 2016 Apr 28;11(4):e0153808. doi: 10.1371/journal.pone.0153808 (PMC4849653; doi:10.1371/journal.pone.0153808)
Supplement: S1 Appendix — (DOCX) [file pone.0153808.s001.docx]

# S1 Appendix. Recording Wildlife Sightings in Denali National Park and Preserve

Observers recorded all sightings of 5 large mammal species: grizzly bear, caribou, Dall’s sheep, moose, and wolves. Data recorded included: date, time, species, and location of sighting. Bus drivers collected written observations on wildlife sightings along the park road from 1995 to 2007 following standard protocols (Tomkiewicz et al. 1999). From 2006 to 2013, touch-screen panels linked to mobile GPS units mounted in buses allowed bus drivers to record and classify wildlife stops along the DPR. Stops were categorized by species (grizzly bear, caribou, Dall Sheep, moose, wolf, or other) and panel entries were georeferenced to locations of the bus (Validator V2000, Universal Tracking, Valencia, CA, USA and Fleet Management System, San Luis Obisbo, CA, USA). Additionally, from 2007-2013, DNPP biological staff members conducted formal surveys of wildlife observed along the park road to validate driver collected data and record additional information on wildlife behavior, number of adults and young, and animal’s distance from the road. From 2007-2009, DNPP biological program staff recorded data on paper forms and from 2010-2013, staff used Juno SB handheld GPS receivers with TerraSync software (Trimble, Sunnyvale, CA, USA) to collect wildlife observation data.

We used two methods to compare data collected by bus drivers and NPS staff in DNPP in 2011. First, we used a proportional population z-test to compare the proportion of trips seeing one or more wolves (probability of sighting) on a trip to Eielson Visitor Center at mile 66 (Fig.3-1). Second, we used a t-test to compare the mean number of groups (individual or more than one) of each species seen by the two methods. The two wolf sighting metrics were not statistically different between two collection methods. The probability of sighting for bus driver data was 0.16 (SE 0.007) and for NPS staff was 0.21 (SE 0.021, χ2=0.62, df=1, P=0.43). The average number of groups of wolves seen based on bus driver data was 0.20 (SE 0.025) and for NPS staff data was 0.34 (SE 0.102, P=0.1728). We therefore combined the data collected by bus drivers from 1997 to 2009 and DNPP staff from 2010-2013 for subsequent analysis.
