# Supplementary figures and images for: Implications of Harvest on the Boundaries of Protected Areas for Large Carnivore Viewing Opportunities
Source: PLoS One. 2016 Apr 28;11(4):e0153808. doi: 10.1371/journal.pone.0153808 (PMC4849653; doi:10.1371/journal.pone.0153808)

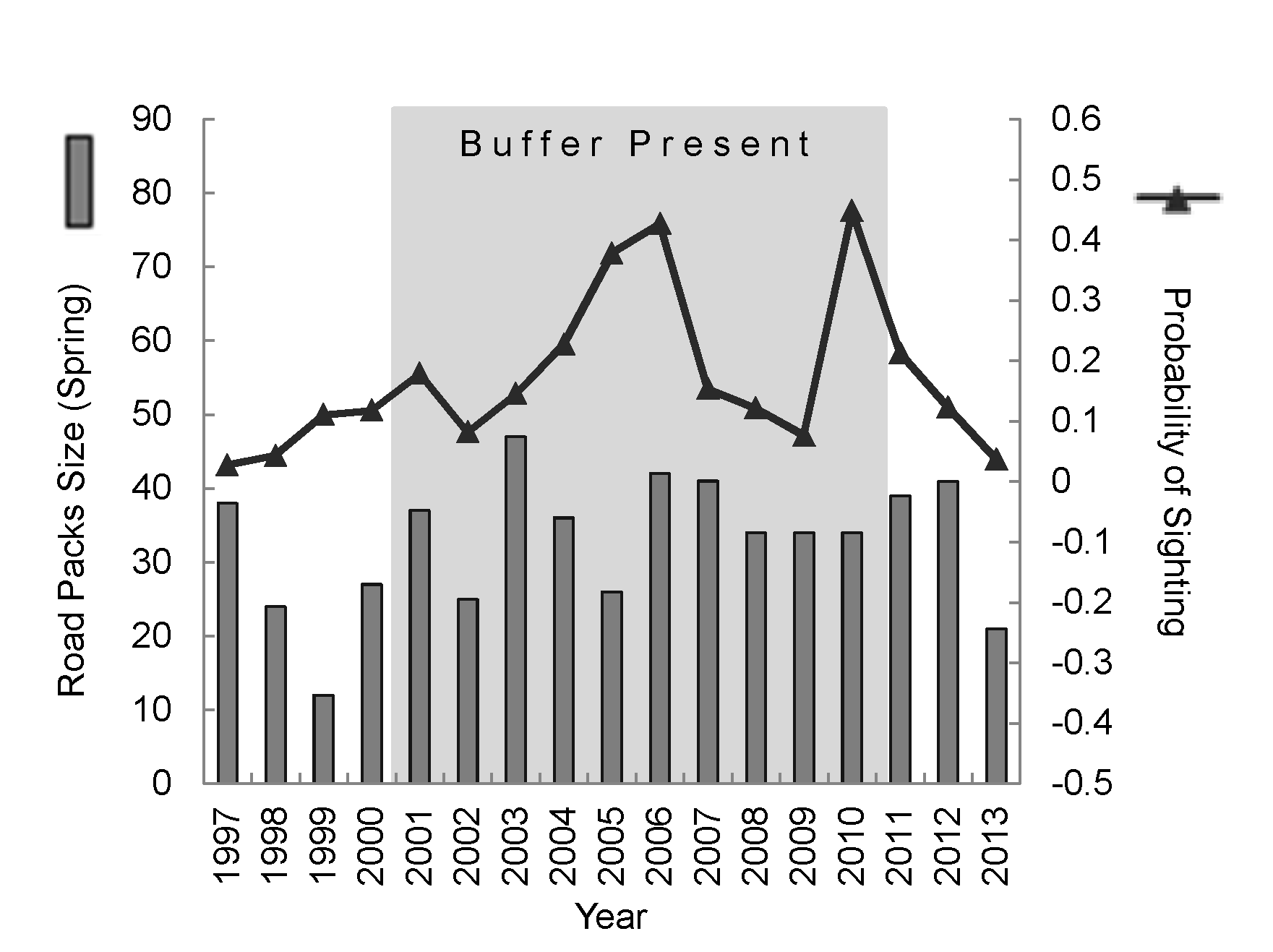

Supplement: S1 Fig — Cumulative count of wolves in road packs in the eastern region of Denali National Park and Preserve (grey bars) and the probability of wolf sightings along the Denali Park Road (black triangles) from 1997 to 2012. Shading indicates years with a harvest buffer zone adjacent to the park in effect. (TIF) [file pone.0153808.s002.tif]

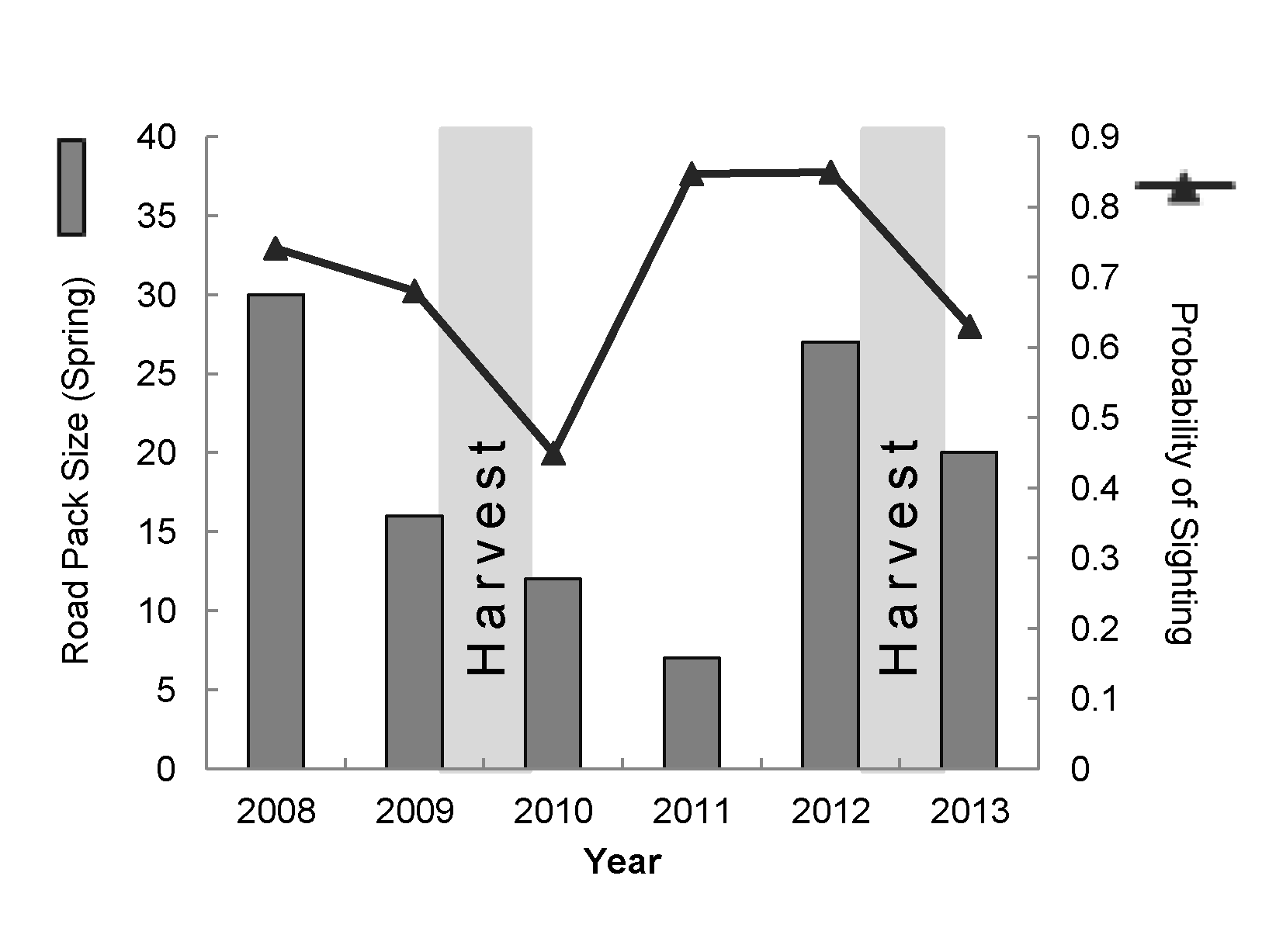

Supplement: S2 Fig — Cumulative count of wolves in road packs in the Northern Range of Yellowstone National Park (grey bars) and probability of wolf sightings in Little America and Lamar Valley (black triangles) from 2008–2012. Hashed bars indicate years preceded by harvest of wolves from road packs. Light gray shading indicates years preceded by harvest of non- pack wolves. (TIF) [file pone.0153808.s003.tif]
