# Supplementary material for: Implications of Harvest on the Boundaries of Protected Areas for Large Carnivore Viewing Opportunities
Source: PLoS One. 2016 Apr 28;11(4):e0153808. doi: 10.1371/journal.pone.0153808 (PMC4849653; doi:10.1371/journal.pone.0153808)
Supplement: S1 Table — Sample size (in number of trips), number of trips with wolf sightings, and annual probability of sighting index for wolves along the Denali Park Road from 1997 to 2013. (DOCX) [file pone.0153808.s004.docx]

**S1 Table. Annual probability of sighting index for Denali National Park and Preserve, Alaska, USA.** Sample size (in number of trips), number of trips with wolf sightings, and annual probability of sighting index for wolves along the Denali Park Road from 1997 to 2013.

| Year | Trips | Trips with Sightings | Annual Probability of Sighting |
| --- | --- | --- | --- |
|  |  |  |  |
| 1997 | 363 | 10 | 0.03 |
| 1998 | 93 | 4 | 0.04 |
| 1999 | 135 | 15 | 0.11 |
| 2000 | 127 | 15 | 0.12 |
| 2001 | 106 | 19 | 0.18 |
| 2002 | 134 | 11 | 0.08 |
| 2003 | 110 | 16 | 0.15 |
| 2004 | 132 | 30 | 0.23 |
| 2005 | 177 | 67 | 0.38 |
| 2006 | 89 | 38 | 0.43 |
| 2007 | 78 | 12 | 0.15 |
| 2008 | 203 | 25 | 0.12 |
| 2009 | 39 | 3 | 0.08 |
| 2010 | 29 | 13 | 0.45 |
| 2011 | 61 | 13 | 0.21 |
| 2012 | 106 | 13 | 0.12 |
| 2013 | 80 | 3 | 0.04 |
