# Supplementary material for: Implications of Harvest on the Boundaries of Protected Areas for Large Carnivore Viewing Opportunities
Source: PLoS One. 2016 Apr 28;11(4):e0153808. doi: 10.1371/journal.pone.0153808 (PMC4849653; doi:10.1371/journal.pone.0153808)
Supplement: S2 Table — Sample size (in number of days within the observation period), number of days with wolf sightings, relative effort for each year (calculated as hours of effort in the given year divided by the maximum number of hours in the field from sampled years), and annual probability of sighting index for wolves in the Lamar Valley and Little America region of Yellowstone National Park from 2008 to 2013. (DOCX) [file pone.0153808.s005.docx]

**S2 Table. Annual probability of sighting index for Yellowstone National Park, Wyoming, USA.** Sample size (in number of days within the observation period), number of days with wolf sightings, relative effort for each year (calculated as hours of effort in the given year divided by the maximum number of hours in the field from sampled years), and annual probability of sighting index for wolves in the Lamar Valley and Little America region of Yellowstone National Park from 2008 to 2013.

| Year | Days in observation period | Days with sightings | Relative Effort | Annual Probability of Sighting |
| --- | --- | --- | --- | --- |
|  |  |  |  |  |
| 2008 | 92 | 91 | 0.75 | 0.74 |
| 2009 | 92 | 66 | 0.95 | 0.68 |
| 2010 | 92 | 48 | 0.86 | 0.45 |
| 2011 | 92 | 78 | 1.00 | 0.85 |
| 2012 | 92 | 84 | 0.93 | 0.85 |
| 2013 | 92 | 69 | 0.84 | 0.63 |
