# Supplementary material for: Implications of Harvest on the Boundaries of Protected Areas for Large Carnivore Viewing Opportunities
Source: PLoS One. 2016 Apr 28;11(4):e0153808. doi: 10.1371/journal.pone.0153808 (PMC4849653; doi:10.1371/journal.pone.0153808)
Supplement: S3 Table — Population size estimates, number of collared wolves, number of collared breeding wolves, and their proportions in the population and harvest included. Population size, number of collared wolves, and number of collared breeders were pre-hunt numbers. (DOCX) [file pone.0153808.s006.docx]

**S3 Table. Summary of wolf harvest for the Eastern Region of Denali National Park and Preserve, Alaska, USA.** Population size estimates, number of collared wolves, number of collared breeding wolves, and their proportions in the population and harvest included**.** Population size, number of collared wolves, and number of collared breeders were pre-hunt numbers.

| Regulatory Year | Fall Population Size | Packs | Collared Wolves | Collared Breeders | Harvest | Collared Harvest | Collared  Breeder Harvest | Proportion of Pop Collared | Proportion Harvest Collared | Diff | Proportion Collared Breeders in Pop | Proportion Collared Breeders in Harvest | Diff |
| --- | --- | --- | --- | --- | --- | --- | --- | --- | --- | --- | --- | --- | --- |
| 1996 | 49 | 6 | 16 | 9 | 4 | 1 | 0 | 0.33 | 0.25 | 0.08 | 0.24 | 0.00 | 0.24 |
| 1997 | 44 | 7 | 22 | 13 | 1 | 1 | 1 | 0.50 | 1.00 | -0.50 | 0.32 | 1.00 | -0.68 |
| 1998 | 35 | 8 | 17 | 11 | 0 | 0 | 0 | 0.49 |  |  | 0.46 |  |  |
| 1999 | 43 | 8 | 17 | 12 | 1 | 1 | 0 | 0.40 | 1.00 | -0.60 | 0.37 | 0.00 | 0.37 |
| 2000 | 49 | 8 | 19 | 13 | 6 | 0 | 0 | 0.39 | 0.00 | 0.39 | 0.33 | 0.00 | 0.33 |
| 2001 | 45 | 9 | 19 | 13 | 1 | 0 | 0 | 0.42 | 0.00 | 0.42 | 0.40 | 0.00 | 0.40 |
| 2002 | 55 | 11 | 14 | 9 | 6 | 0 | 0 | 0.25 | 0.00 | 0.25 | 0.40 | 0.00 | 0.40 |
| 2003 | 55 | 9 | 13 | 9 | 9 | 6 | 5 | 0.24 | 0.67 | -0.43 | 0.33 | 0.56 | -0.23 |
| 2004 | 49 | 9 | 12 | 7 | 3 | 1 | 1 | 0.24 | 0.33 | -0.09 | 0.37 | 0.33 | 0.03 |
| 2005 | 77 | 9 | 13 | 4 | 3 | 1 | 1 | 0.17 | 0.33 | -0.16 | 0.23 | 0.33 | -0.10 |
| 2006 | 75 | 9 | 18 | 5 | 9 | 1 | 0 | 0.24 | 0.11 | 0.13 | 0.24 | 0.00 | 0.24 |
| 2007 | 84 | 10 | 22 | 11 | 11 | 3 | 2 | 0.26 | 0.27 | -0.01 | 0.24 | 0.18 | 0.06 |
| 2008 | 56 | 9 | 17 | 12 | 8 | 2 | 1 | 0.30 | 0.25 | 0.05 | 0.32 | 0.13 | 0.20 |
| 2009 | 57 | 8 | 12 | 9 | 11 | 1 | 1 | 0.21 | 0.09 | 0.12 | 0.28 | 0.09 | 0.19 |
| 2010 | 53 | 6 | 12 | 7 | 3 | 1 | 0 | 0.23 | 0.33 | -0.11 | 0.23 | 0.00 | 0.23 |
| 2011 | 45 | 5 | 13 | 9 | 3 | 1 | 1 | 0.29 | 0.33 | -0.04 | 0.22 | 0.33 | -0.11 |
| 2012 | 29 | 4 | 10 | 7 | 2 | 0 | 0 | 0.34 | 0.00 | 0.34 | 0.28 | 0.00 | 0.28 |
