# Supplementary material for: Implications of Harvest on the Boundaries of Protected Areas for Large Carnivore Viewing Opportunities
Source: PLoS One. 2016 Apr 28;11(4):e0153808. doi: 10.1371/journal.pone.0153808 (PMC4849653; doi:10.1371/journal.pone.0153808)
Supplement: S5 Table — Population size estimates, number of collared wolves, number of collared breeding wolves, and their proportions in the population and harvest included. Population size and number of collared wolves were pre-hunt numbers. (DOCX) [file pone.0153808.s008.docx]

**S5 Table. Summary of wolf harvest for wolf packs in Yellowstone National Park, Wyoming, USA.** Population size estimates, number of collared wolves, number of collared breeding wolves, and their proportions in the population and harvest included. Population size and number of collared wolves were pre-hunt numbers.

| Regulatory Year | Fall  Population Size | Packs | Collared Wolves | Harvest | Collared Harvest | Proportion of Pop Collared | Proportion Harvest Collared | Diff |
| --- | --- | --- | --- | --- | --- | --- | --- | --- |
| 2009 | 128 | 14 | 36 | 4 | 2 | 0.28 | 0.50 | -0.22 |
| 2011 | 135 | 11 | 31 | 2 | 1 | 0.23 | 0.50 | -0.27 |
| 2012 | 99 | 10 | 27 | 12 | 7 | 0.27 | 0.58 | -0.31 |
