# Supplementary material for: Implications of Harvest on the Boundaries of Protected Areas for Large Carnivore Viewing Opportunities
Source: PLoS One. 2016 Apr 28;11(4):e0153808. doi: 10.1371/journal.pone.0153808 (PMC4849653; doi:10.1371/journal.pone.0153808)
Supplement: S6 Table — Candidate model set includes the factor RoadPop. K is the number of parameters in the model, PNRI is the Pack Near Road Index, TotalPop is the wolf population size, RoadPop is the number of wolves in packs that overlap the Denali Park Road, Buffer is a factor indicating the presence/absence of a harvest buffer, WolfHarv is the number of wolves harvested in the prior year and BreedHarv is a binary factor describing if breeders were or were not harvested from road packs in the prior year. (DOCX) [file pone.0153808.s009.docx]

**S6 Table. Model selection table evaluating factors potentially affecting probability of wolf sightings in Denali National Park and Preserve, Alaska, USA (including the factor RoadPop).** Candidate model set includes the factor RoadPop. K is the number of parameters in the model, PNRI is the Pack Near Road Index, TotalPop is the wolf population size, RoadPop is the number of wolves in packs that overlap the Denali Park Road, Buffer is a factor indicating the presence/absence of a harvest buffer, WolfHarv is the number of wolves harvested in the prior year and BreedHarv is a binary factor describing if breeders were or were not harvested from road packs in the prior year.

| Model | K | AICc | ΔAICc | Model Likelihood | AICc Weight | Log Liklihood | Pseudo R^2^ |
| --- | --- | --- | --- | --- | --- | --- | --- |
| PackNearRoad+Buffer+WolfHarv | 4 | 154.34 | 0.00 | 1.00 | 1.00 | -71.50 | 0.67 |
| PackNearRoad+Buffer | 3 | 175.72 | 21.39 | 0.00 | 0.00 | -83.94 | 0.55 |
| PackNearRoad+Buffer+BreedHarv | 4 | 178.47 | 24.13 | 0.00 | 0.00 | -83.57 | 0.55 |
| TotalPop+Buffer+WolfHarv | 4 | 187.78 | 33.44 | 0.00 | 0.00 | -88.22 | 0.51 |
| TotalPop+Buffer+BreedHarv | 4 | 188.19 | 33.85 | 0.00 | 0.00 | -88.43 | 0.51 |
| PackNearRoad+WolfHarv | 3 | 189.54 | 35.20 | 0.00 | 0.00 | -90.85 | 0.48 |
| TotalPop+Buffer | 3 | 196.43 | 42.09 | 0.00 | 0.00 | -94.29 | 0.45 |
| PackNearRoad | 2 | 198.63 | 44.30 | 0.00 | 0.00 | -96.89 | 0.43 |
| PackNearRoad+BreedHarv | 3 | 199.70 | 45.36 | 0.00 | 0.00 | -95.93 | 0.44 |
| RoadPop+Buffer+BreedHarv | 4 | 209.64 | 55.31 | 0.00 | 0.00 | -99.15 | 0.41 |
| Buffer | 2 | 212.27 | 57.94 | 0.00 | 0.00 | -103.71 | 0.36 |
| TotalPop+BreedHarv | 3 | 213.99 | 59.66 | 0.00 | 0.00 | -103.07 | 0.37 |
| RoadPop+Buffer+WolfHarv | 4 | 214.70 | 60.36 | 0.00 | 0.00 | -101.68 | 0.38 |
| RoadPop+Buffer | 3 | 215.23 | 60.90 | 0.00 | 0.00 | -103.69 | 0.36 |
| TotalPop | 2 | 238.72 | 84.38 | 0.00 | 0.00 | -116.93 | 0.24 |
| TotalPop+WolfHarv | 3 | 240.84 | 86.51 | 0.00 | 0.00 | -116.50 | 0.24 |
| RoadPop+BreedHarv | 3 | 264.62 | 110.29 | 0.00 | 0.00 | -128.39 | 0.13 |
| WolfHarv | 2 | 284.58 | 130.25 | 0.00 | 0.00 | -139.86 | 0.02 |
| RoadPop+WolfHarv | 3 | 287.57 | 133.23 | 0.00 | 0.00 | -139.86 | 0.02 |
| RoadPop | 2 | 288.00 | 133.67 | 0.00 | 0.00 | -141.57 | 0.01 |
